# Involvement of gonadotropin-inhibitory hormone in pubertal disorders induced by thyroid status

**DOI:** 10.1038/s41598-017-01183-8

**Published:** 2017-04-21

**Authors:** Mika Kiyohara, You Lee Son, Kazuyoshi Tsutsui

**Affiliations:** 1 Laboratory of Integrative Brain Sciences, Department of Biology, Waseda University, and Center for Medical Life Science of Waseda University, Shinjuku-ku, Tokyo 162-8480 Japan; 2 Department of Pediatrics, The Jikei University School of Medicine, Minato-ku Tokyo, 105-8461 Japan

## Abstract

Thyroid disorders cause abnormal puberty, indicating interactions between the hypothalamus-pituitary-thyroid (HPT) and hypothalamus-pituitary-gonadal (HPG) axes, which are important in pubertal development. The hypothalamic gonadotropin-inhibitory hormone (GnIH) was shown to be decreased in the early prepubertal stage, suggesting the role of GnIH on pubertal onset. Here, we investigated whether thyroid dysfunction affects pubertal onset in female mice *via* GnIH regulation. Hypothyroidism showed delayed pubertal onset with increased GnIH expression and reduced pituitary-gonadal activity. Remarkably, knockout of GnIH prevented the effect of hypothyroidism to delay the pubertal onset, resulting in indistinguishable pubertal timing in GnIH-knockout female mice between control and hypothyroidism-induced group, indicating that increased GnIH expression induced by hypothyroidism may lead to delayed puberty. In contrast, hyperthyroidism led to a decrease in GnIH expression, however pubertal onset was normal, implying further reduction of the inhibitory GnIH had little effect on the phenotypical change. Critically, thyroid hormone suppressed GnIH expression in hypothalamic explants and GnIH neurons expressed thyroid hormone receptors to convey the thyroid status. Moreover, the thyroid status highly regulated the chromatin modifications of *GnIH* promoter, H3acetylation and H3K9tri-methylation. These findings indicate a novel function of GnIH to mediate HPT-HPG interactions that contribute to proper pubertal development.

## Introduction

Thyroid hormones (THs; thyroxine, T_4_ and triiodothyronine, T_3_), known as regulators of metabolism, development and growth^1–3^, play an important role in proper development and function of the reproductive system, particularly in pubertal onset^4, 5^. It was shown that conversion of T_4_ to bioactive T_3_ is increased with entry into puberty^6^, thus delayed pubertal onset is often observed clinically in children with hypothyroidism; sometimes, precocious puberty in case of extreme hypothyroidism^7–9^. Pubertal onset is regulated in part by a brain-dependent process, whereby increased pulsatile secretion of hypothalamic gonadotropin-releasing hormone (GnRH) leads to the activation of pituitary-gonadal axis to awake the entire reproductive system^10^. There are some explanations on how abnormal thyroid status leads to pubertal disorders based on the multilevel interactions of the two neuroendocrine systems, the hypothalamus-pituitary-thyroid (HPT) axis and the hypothalamus-pituitary-gonadal (HPG) axis. First, elevated levels of thyrotropin-releasing hormone in hypothyroidism induce hyperprolactinemia and alter GnRH pulsatile secretion, which lead to a delay in luteinizing hormone (LH) response, thus result in delayed puberty^11^. Second, increased thyroid-stimulating hormone (TSH) levels activate gonadal function by stimulating follicle-stimulating hormone (FSH) receptor expressed in gonads, because the structure of FSH and TSH receptors is very similar, which is responsible for precocious puberty^12, 13^. Although some mediators in HPT-HPG interaction have been suggested^11–13^, the mechanism underlying how TH acts on the HPG axis has not been fully elucidated. Additionally, studies on the effect of abnormal thyroid status, hypothyroidism and hyperthyroidism, on reproductive development have produced conflicting results^14, 15^. Parallel studies that compare the effects of hypothyroidism and hyperthyroidism on pubertal onset *via* HPG regulation have a potential to answer these questions.

Gonadotropin-inhibitory hormone (GnIH, also known as RFamide-related peptide, RFRP) is a newly discovered hypothalamic neuropeptide that actively inhibits gonadotropin secretion, which was first identified in the Japanese quail^16^. GnIH is conserved across mammals, including mice, rat and humans, and its function to inhibit gonadotropin secretion is also demonstrated^17–22^, indicating the role of GnIH as a negative regulator of reproduction. Inhibitory effect of GnIH on reproduction is mainly accomplished at hypothalamic-pituitary levels^23^. Cell bodies of GnIH neurons are located in the dorsomedial hypothalamic area (DMH) in most mammals^24–26^. The fibers of GnIH neurons contact with GnRH neurons that express GnIH receptors (GnIH-Rs; GPR147 and GPR74)^18, 19, 27, 28^, and the direct suppressive effects of GnIH on GnRH neurons in their neuronal activity and firing rate of GnRH neurons, as well as GnRH release^29–31^ have also been verified. GnIH may also act on kisspeptin neurons. Kisspeptin, the product of *Kiss1* gene, is a potent stimulator of GnRH/gonadotropin secretion by two modes of pulse and surge to regulate puberty onset and normal reproductive performance^32^. It has been shown that a subset of kisspeptin neurons expresses GnIH-Rs and receives GnIH fiber contact^33^, suggesting the possible inhibitory role of GnIH in the regulation of kisspeptin neurons. In addition to the effect of GnIH on hypothalamic neurons, GnIH neurons also project to the median eminence to control anterior pituitary function *via* GnIH-Rs expressed in gonadotropes^18, 19, 34^. Although there is some debate whether GnIH can directly act on the pituitary in some species, GnIH decreases the synthesis and/or release of pituitary gonadotropins, LH and FSH in many species^27, 35–37^. Together, these findings suggest that GnIH is a key regulatory factor of the HPG axis to govern the neuronal activities of GnRH and kisspeptin, and eventually gonadotropin secretion. From a developmental standpoint, both GnIH expression and neuronal activation decreased markedly in the early prepubertal stage in the dorsomedial hypothalamic nucleus of female mice^38, 39^, when the pulsatile GnRH secretion was increased after a quiescent period during infancy. Thus, decreased GnIH levels in this period may be interpreted by the removal of an inhibitory factor involved in quiescent state of GnRH neurons to initiate puberty.

The present experiments were designed to determine the possible role of GnIH in reproductive dysfunction induced by abnormal thyroid status, focusing on the timing of pubertal onset. To do this, we induced hypothyroidism and hyperthyroidism in juvenile female mice by long-term administration of propylthiouracil (PTU) and T_4_, respectively, then analyzed the change in the regulatory factors of the HPG axis; GnIH, Kiss1, GnRH, LH, and estradiol (E2).

## Results

### Hypothyroidic female mice showed delayed pubertal onset and increased GnIH expression

**Figure 1:**
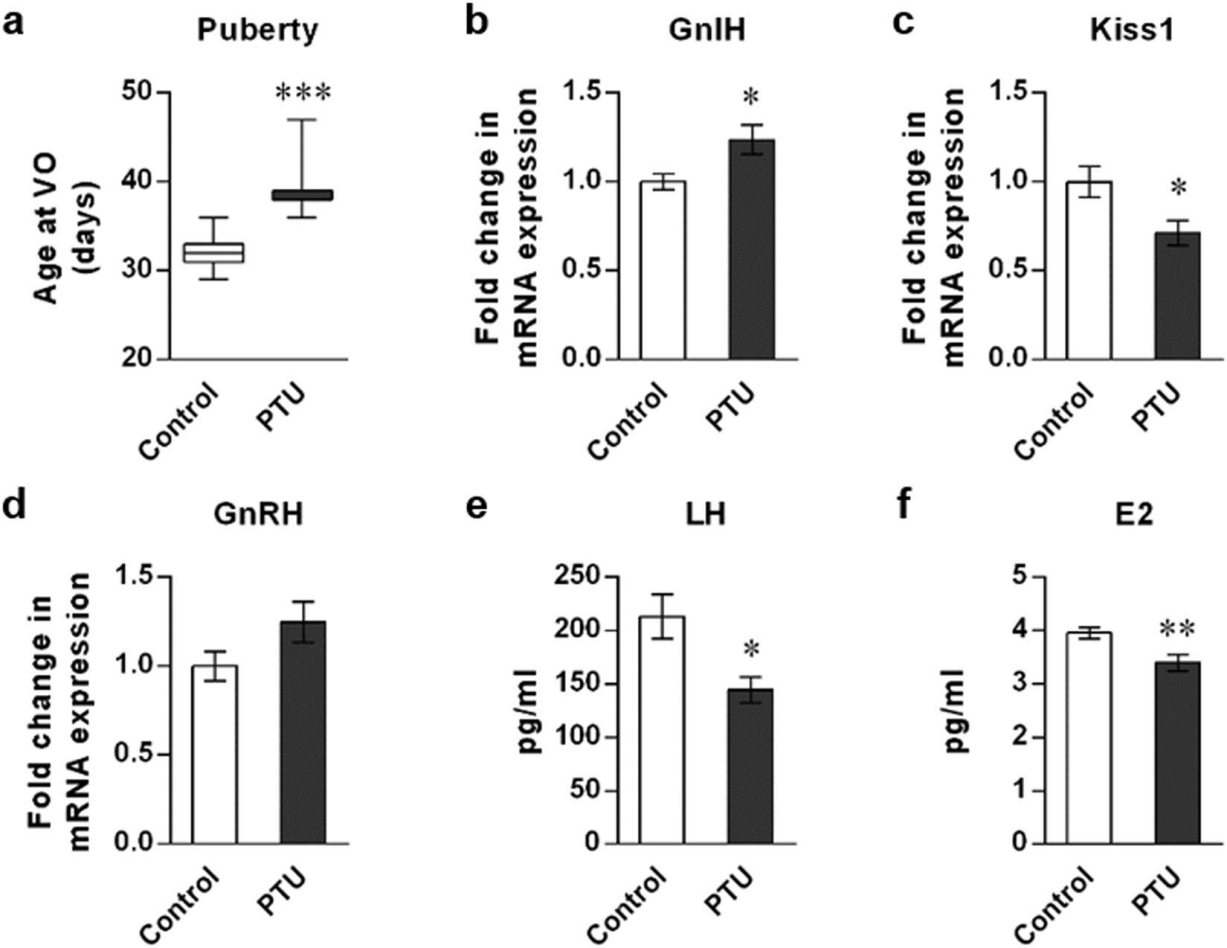
Effect of hypothyroidism on puberty onset and the reproductive markers of the HPG axis in female mice. (**a**) Effect of PTU administration on puberty onset. Female mice aged 20 days were induced to hypothyroidism by 0.1% PTU administration in drinking water. The timing of puberty was assessed by monitoring vaginal opening (VO). Data are shown as the box-and-whisker plots with the smallest value and up to the largest (*Min* to *Max*). To measure the changes in HPG markers, brain and blood samples were collected at day 34. (**b**–**d**) Gene expression change in the hypothalamic regulators, GnIH (**b**), Kiss1 (**c**) and GnRH (**d**). mRNA levels were determined by RT-qPCR relative to the reference gene GAPDH. (**e**,**f**) Changes in LH (**e**) and E2 (**f**) levels were measured in serum samples. Each column and vertical line represents mean ± SEM. (**a**) n = 10 in control and n = 9 in PTU group; (**b–d**) n = 7 per group; (**e**) n = 12 in control and n = 9 in PTU group; (**f**) n = 13 in control and n = 14 in PTU group). *p < 0.05; **p < 0.01; ***p < 0.001 by unpaired *t* test.

Immature female mice 20 days after birth were subjected to the long-term hypothyroid status by 0.1% PTU administration in drinking water. Successful induction of hypothyroidism was confirmed by low T_3_ and elevated TSH levels through negative feedback loop, and loss of weight (Supplementary Fig. 1A–C). Pubertal onset was significantly delayed in hypothyroidic mice compared to control mice (39.3 ± 1.0 *vs*. 32.1 ± 0.6 days; p < 0.001; Fig. 1a), indicating that hypothyroid status could negatively impact pubertal development. In hypothyroid status, hypothalamic GnIH mRNA expression was significantly increased (p < 0.05; Fig. 1b), whereas Kiss1 was decreased (p < 0.05; Fig. 1c). We did not find significant differences in GnRH mRNA expression in hypothyroidic mice (Fig. 1d). Downstream effectors of hypothalamic regulation, serum LH and E2 levels were also decreased (p < 0.05; LH in Fig. 1e, p < 0.01; E2 in Fig. 1f). These results indicate that each stage of the HPG axis besides GnRH mRNA is directed to delay the timing of pubertal onset, and increased GnIH mRNA expression may lead to the reduction of positive regulators of the HPG axis, Kiss1 mRNA, LH, and E2.

### Knockout of GnIH prevents the delayed pubertal onset induced by hypothyroidism

**Figure 2:**
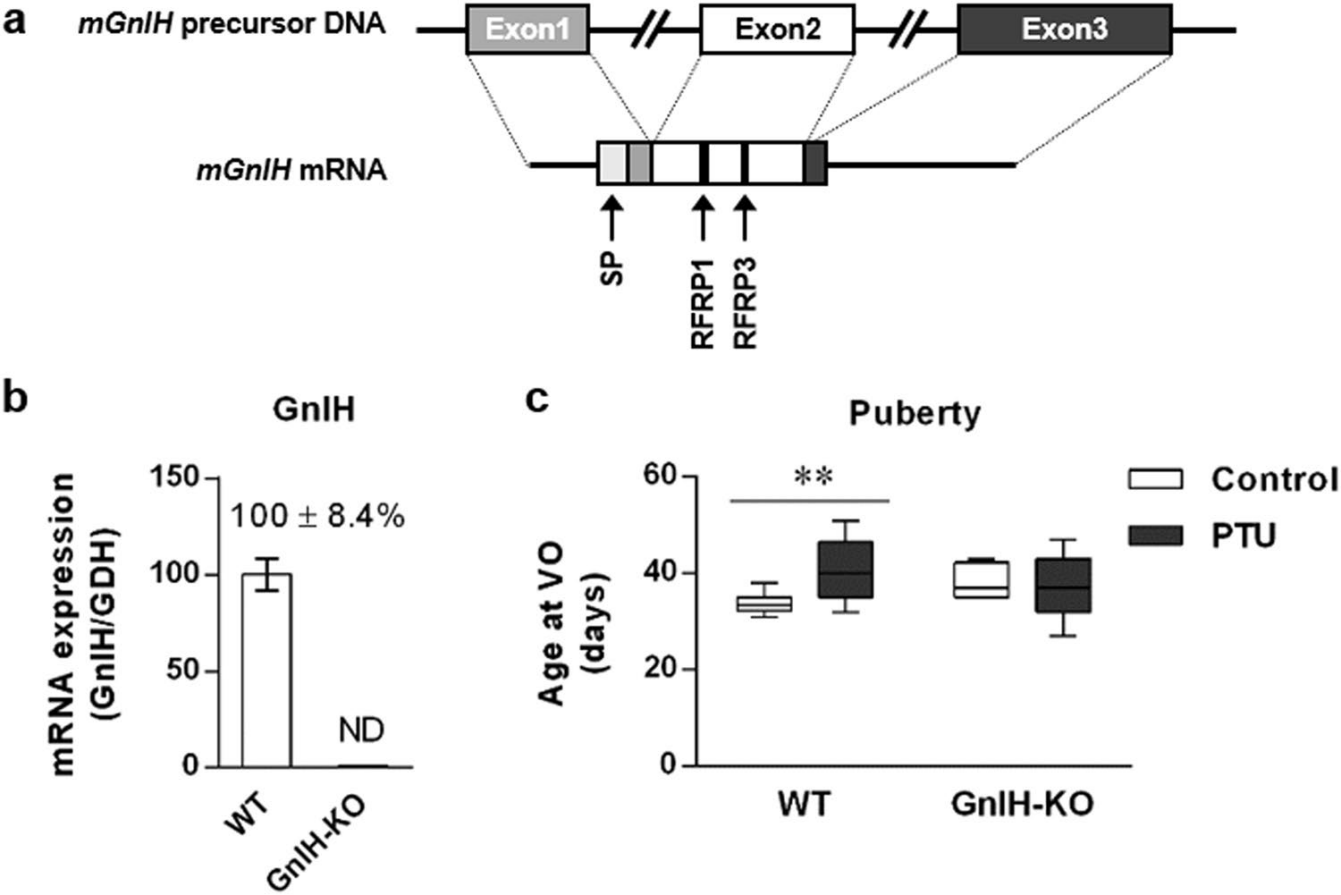
Effect of hypothyroidism on pubertal onset in GnIH-KO female mice. (**a**) Schematic diagram of the GnIH precursor DNA and mRNA. GnIH-KO mice were generated by deleting the exon 2 region encoding two kinds of putative mouse GnIH peptides, RFRP1 and 3. SP, signal peptide. (**b**) GnIH mRNA levels in the hypothalamus of WT or GnIH-KO female mice. GnIH mRNA was not detected in GnIH-KO mice. ND, not detected. (**c**) WT or GnIH-KO mice aged 20 days were induced to hypothyroidism by 0.1% PTU administration, and the timing of pubertal onset was assessed as described in Fig. 1a. Data are shown as the box-and-whisker plots with *Min* to *Max* (**c**) n = 8 in control and n = 17 in PTU of WT group; n = 10 in control and n = 15 in PTU of GnIH-KO group). **p < 0.01 by two-way ANOVA Bonferroni post-test, ANOVA summary (interaction, *F*_*1*,*46*_ = 6.711, p = 0.0128; effect for GnIH expression, *F*_*1*,*46*_ = 0.08374, p = 0.7736; effect for PTU administration, *F*_*1*,*46*_ = 3.4934, p = 0.0680).

We hypothesized that the reason why pubertal onset is delayed by hypothyroidism is that the inhibitory factor GnIH is not decreased during this period. To verify this, we induced hypothyroidism into GnIH-knockout (KO) female mice. GnIH-KO mice were generated by deletion of the exon 2 in GnIH precursor gene encoding GnIH peptide^40^ (Fig. 2a), and GnIH mRNA levels were not detected in the hypothalamus of GnIH-KO mice (Fig. 2b). Although 0.1% PTU-administration effectively reduced serum T_3_ levels by 57% in GnIH-KO mice compared to control (Supplementary Fig. 2A), there were no significant differences in body weight between PTU and control of GnIH-KO mice (Supplementary Fig. 2B). Importantly, hypothyroidism-induced delayed puberty as observed in WT was blocked in PTU-administered GnIH-KO female mice, leading to an equivalent pubertal onset to control GnIH-KO mice (Fig. 2c, p < 0.01 in WT mice, 33.9 ± 1.5 (Control) *vs*.40. 9 ± 0.8 (PTU) days, and no significant difference in GnIH-KO mice, 38.4 ± 1.6 (Control) *vs*. 37.3 ± 1.6 (PTU) days). These results indicate that the negative effect of hypothyroidism on pubertal development is alleviated by knockout of GnIH in support of our hypothesis of which increased GnIH levels may contribute to delay pubertal onset in hypothyroidism state.

### Hyperthyroidic female mice showed decreased GnIH expression

**Figure 3:**
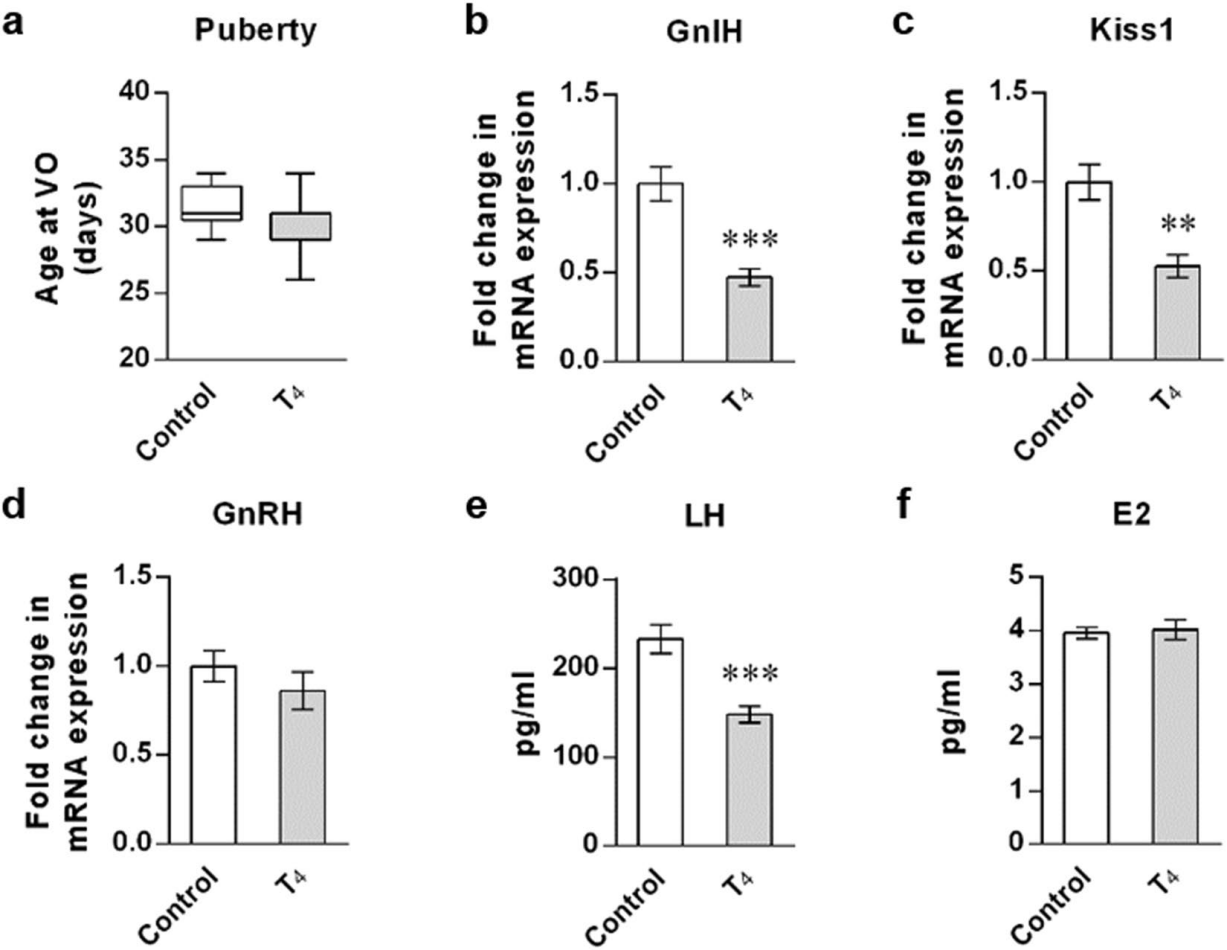
Effect of hyperthyroidism on puberty onset and the reproductive markers of the HPG axis in female mice. (**a**) Effect of T_4_ administration on puberty onset. Female mice aged 20 days were induced to hyperthyroidism by 0.001% T_4_ administration in drinking water. The timing of puberty was assessed as described in Fig. 1a. Data are shown as the box-and-whisker plots with *Min* to *Max*. (**b**–**d**) Gene expression change in the hypothalamic GnIH (**b**), Kiss1 (**c**) and GnRH (**d**). mRNA levels were determined by RT-qPCR as described in Fig. 1b–d. (**e**,**f**) Changes in LH (**e**) and E2 (**f**) levels were measured in serum samples. Each column and vertical line represents mean ± SEM (**a**) n = 18 in control and n = 23 in T_4_ group; (**b–d**) n = 6 in control and n = 7 in T_4_ group; (**e**) n = 12 in control and n = 14 in T_4_ group; (**f**) n = 13 in control and n = 14 in T_4_ group). **p < 0.01; ***p < 0.001 by unpaired *t* test.

In contrast to abnormal pubertal onset shown in hypothyroidic mice, hyperthyroidism induced by 0.001% T_4_ administration had no effect on pubertal onset in female mice (Fig. 3a), although we confirmed the significant changes in circulating T_3_ and TSH levels, as well as body weight (Supplementary Fig. 1D–F). In hyperthyroidic mice, there were complicated changes in the HPG axis. Both of hypothalamic GnIH and Kiss1 mRNA expressions were decreased (p < 0.001; Fig. 3b, p < 0.01; Fig. 3c), whereas GnRH mRNA expression was not changed (Fig. 3d). Although LH release was significantly reduced in hyperthyroidic mice (p < 0.001; Fig. 3e), there was no change in E2 levels (Fig. 3f), reflecting no differences in pubertal onset between hyperthyroidic and control mice.

### GnIH expression is suppressed by high concentration of T_3_

**Figure 4:**
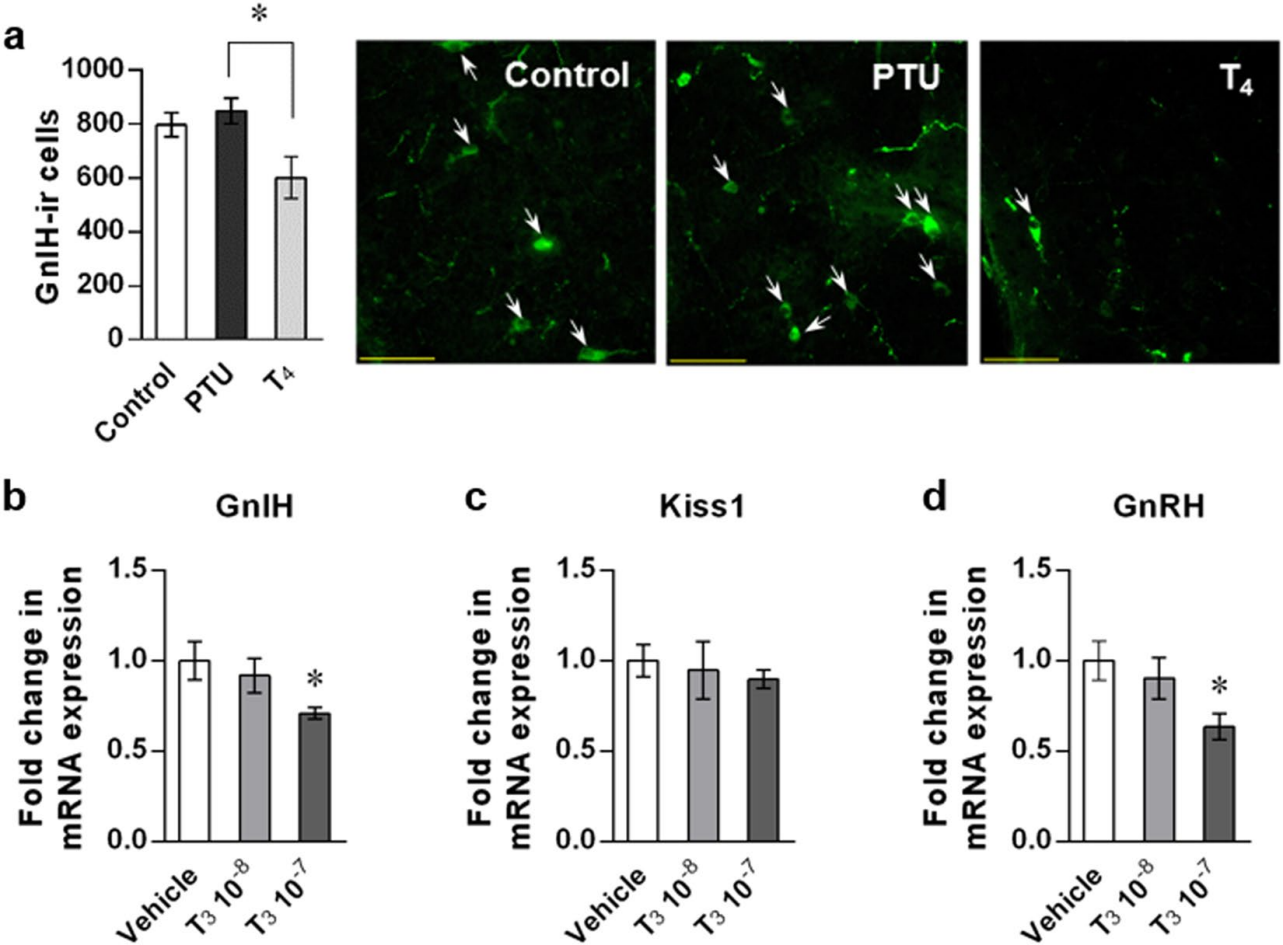
High concentration T_3_ suppress GnIH expression. (**a**) Effect of hypothyroidism and hyperthyroidism on GnIH-ir cell numbers in the DMH. GnIH-ir cells were counted in the DMH of control, PTU-induced hypothyroidic or T_4_-induced hyperthyroidic mice. The delayed pubertal onset was confirmed in PTU group. Data represent mean ± SEM (n = 5 in control and T_4_ group, and n = 4 in PTU group). *p < 0.05; PTU *vs*. T_4_ by one-way ANOVA Tukey’s post-test, ANOVA summary (*F*_*2*,*11*_ = 4.636, p = 0.0347). Representative images of GnIH-ir cells in control, PTU or T_4_-administered mice are shown. Scale bar indicates 50 μm. (**b**–**d**) Effect of T_3_ treatment on hypothalamic GnIH (**b**), Kiss1 (**c**) and GnRH (**d**) mRNA expression. Hypothalamic explants from female mice aged 20 days were treated with vehicle or the indicated concentrations of T_3_ (10^−8^ and 10^−7^ M) for 3 h. mRNA levels were determined by RT-qPCR relative to the reference gene GAPDH. Data represent mean ± SEM (n = 6 per group). (**b**) *p = 0.0269; Vehicle *vs*. T_3_ 10^−7^ by one-way ANOVA by uncorrected Fisher’s LSD post-test, ANOVA summary (*F*_*2*,*14*_ = 3.356, p = 0.0645). (**d**) *p* = *0.0202; Vehicle *vs*. T_3_ 10^−7^ by one-way ANOVA by uncorrected Fisher’s LSD post-test, ANOVA summary (*F*_*2*,*15*_ = 3.6282, p = 0.0519).

The change of GnIH mRNA expression was negatively correlated with the thyroid status as shown in Figs 1b and 3b, and the number of GnIH-positive cells in the DMH was decreased in T_4_-induced hyperthyroidic mice compare to PTU-induced hypothyroidic mice (p < 0.05; Fig. 4a). These results indicate that high concentration of T_3_ may suppress GnIH expression. To test this, we examined the direct effect of T_3_ on GnIH expression in hypothalamic explants. GnIH mRNA levels were significantly decreased by 10^−7^ M of T_3_ treatment in female hypothalamic samples, consistent with hyperthyroidic mice (Fig. 4b, p = 0.0269 by one-way ANOVA followed by uncorrected Fisher’s least significand difference (LSD) test). On the contrary, the results of Kiss1 and GnRH mRNA expressions were not as expected; decreased Kiss1 expression shown in hyperthyroidic mice was not observed in hypothalamic explants (Fig. 4c), and GnRH expression was reduced in hypothalamic explants, despite of no changes in hyperthyroidic mice (Fig. 4d, p = 0.0202 by one-way ANOVA followed by uncorrected Fisher’s LSD test). Although there are some inconsistencies between hyperthyroidic mice and T_3_-treated hypothalamic explants, these results reinforce the inhibitory effect of T_3_ on GnIH mRNA expression.

### Hypothalamic GnIH neurons express TRα and TRβ

**Figure 5:**
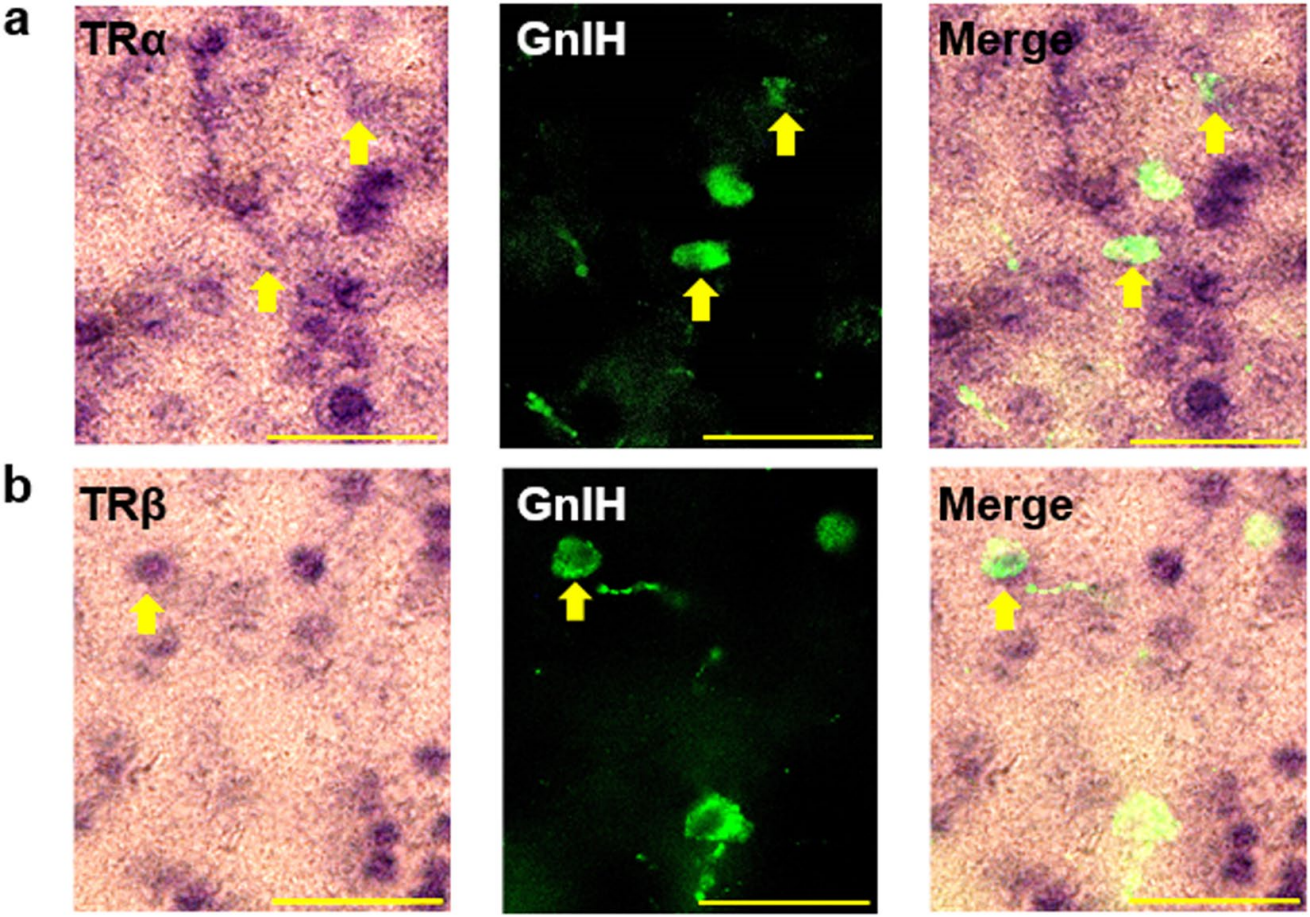
GnIH neurons express TRα and TRβ in the DMH. (**a**,**b**) Double staining of *in situ* hybridization (ISH) for TRα (**a**) or TRβ (**b**) and immunohistochemistry (IHC) for GnIH-ir cells were performed with female mice aged 20 days, and the merged image of ISH and IHC was shown. Arrows indicate GnIH cells expressing TRα (**a**) or TRβ (**b**). Scale bar indicates 50 μm.

Thyroid status altered hypothalamic GnIH expression *in vivo*, and T_3_-induced reduction of GnIH mRNA levels in hypothalamic explants was also observed. Thus, we tested whether hypothalamic GnIH neurons express TH receptors (TRs) that allow a direct response to TH. By double labeling for TRα or TRβ with GnIH, we found that GnIH-immunoreactive (ir) cells express both TRα and TRβ mRNA (Fig. 5a and b), showing that TRα or TRβ is co-labeled with GnIH neurons in a ratio of 51.1 ± 6.0% or 33 ± 2.9%, respectively. These results indicate that TRs may convey TH signals directly to GnIH neurons.

### Epigenetic modification of *GnIH* promoter region is changed depending on the thyroid status in female mice

**Figure 6:**
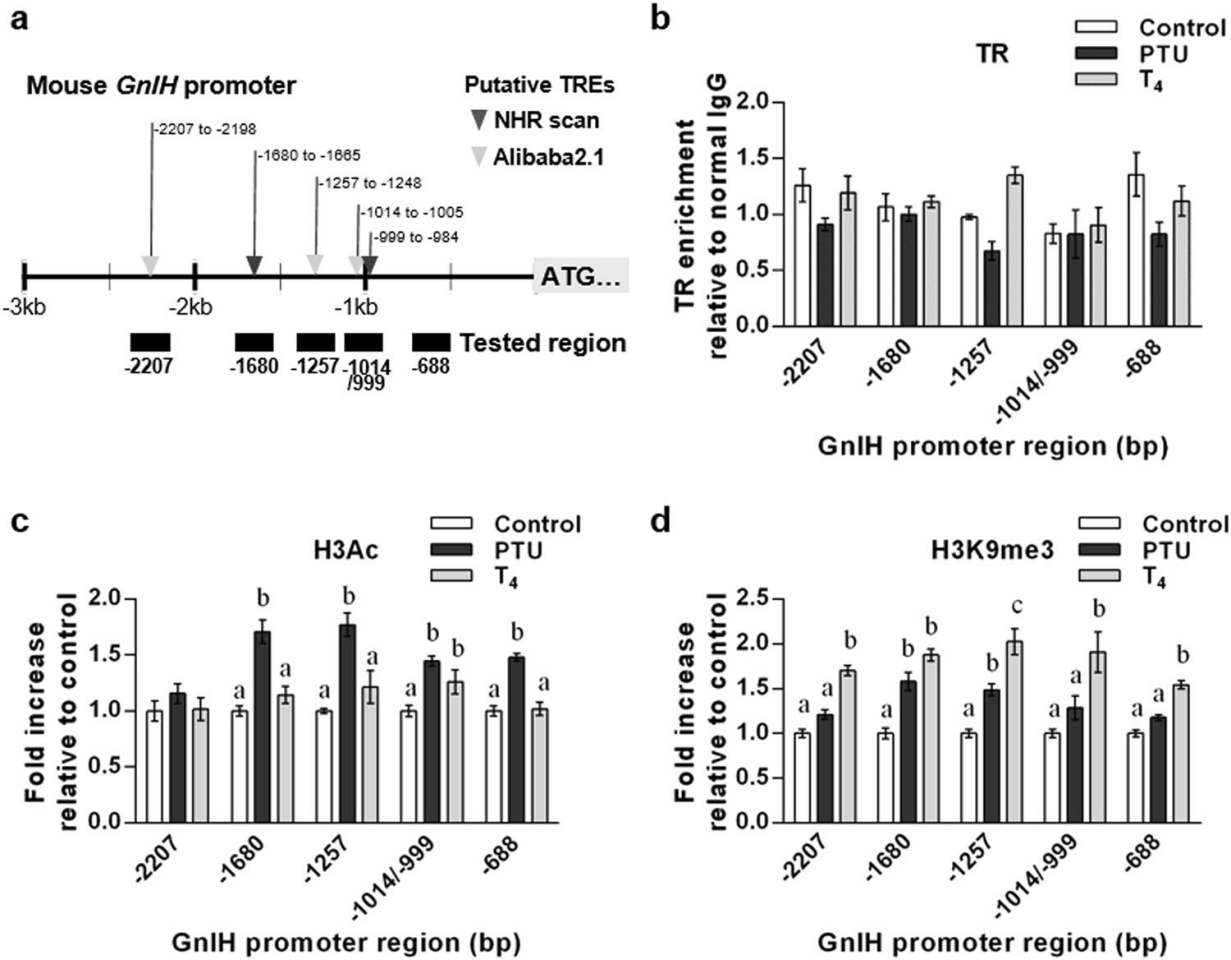
Chromatin modification status of the 5’flanking region of *GnIH* gene in hypothyroidic and hyperthyroidic female mice. (**a**) Schematic diagram of the promoter region of *GnIH* gene. Putative TREs predicted by NHR scan (▼) and AliBaba 2.1 () are indicated at their locations from ORF. (**b–d**) ChIP-qPCR was performed to scan TR occupancy and chromatin modifications across the *GnIH* promoter region. Chromatin samples from control, PTU-induced hypothyroidic or T_4_-induced hyperthyroidic female mice were precipitated using specific antibodies for TR (**b**), acetylation of histone H3 (H3Ac, (**c**)) and histone H3-Lysine 9 tri-methylation (H3K9me3, (**d**). Normal rabbit IgG was used as a negative control for the specificity of immunoprecipitation. TR occupancy in (**b**) is expressed as relative enrichment with normal IgG signal. Levels of histone modification in (**c**,**d**) are normalized to total histone H3 and expressed as fold increase relative to control. Data represent mean ± SEM (**(b)**, n = 3 per group; n = 1 is 5 hypothalamic samples; (**c**,**d**) n = 6 per group). Letters indicate significant differences, p < 0.05 by two-way ANOVA Bonferroni post-test. (**c**) H3Ac, ANOVA summary; interaction, *F*_*8*,*75*_ = 2.809, p = 0.0089; effect for promoter region, *F*_*4*,*75*_ = 5.005, p = 0.0012; effect for thyroid status, *F*_*2*,*75*_ = 52.48, p < 0.001. (**d**) H3K9me3, ANOVA summary; interaction, *F*_*8*_,_*75*_ = 1.628, p = 0.1312; effect for promoter region, *F*_*4*_,_*75*_ = 4.488, p = 0.0026; effect for thyroid status, *F*_*2*_,_*75*_ = 95.46, p < 0.001.

There are several putative TH-response elements (TREs) within 3 kb upstream region from the mouse *GnIH* ORF (Fig. 6a; predicted by using the prediction programs; NHR scan, http://www.cisreg.ca/cgi-bin/NHR-scan/nhr_scan.cgi and Alibaba2.1, http://www.gene-regulation.com/pub/programs/alibaba2/index.html). To determine whether TRs directly act on the promoter region of *GnIH* to regulate its expression, we performed chromatin immunoprecipitation (ChIP) assays to monitor TR recruitment on the *GnIH* promoter and the change in chromatin modifications by the thyroid status. Although direct binding of TRs on the tested *GnIH* promoter region was not observed (Fig. 6b), histone H3 modification status was actively changed by TH levels in female mice. H3 acetylation (H3Ac), which is tightly associated with gene activation, was significantly increased in hypothyroidic mice at −1680, −1257 and −688 bp region from the ORF, compared to control and hyperthyroidic mice (Fig. 6c). Reversely, H3 lysine 9 tri-methylation (H3K9me3), which is correlated with transcriptional repression, was most enriched in hyperthyroidic mice at −2207, −1257, −1014/999 and −688 bp compared to control and hypothyroidic mice (Fig. 6d). Together, these results suggest that chromatin modification of the *GnIH* promoter region is highly regulated by thyroid status consistent with its gene expression change, whereas this regulation does not require the direct binding of TRs to the *GnIH* promoter.

## Discussion

Thyroid disorders lead to abnormal pubertal development. Although interactions between the HPT and HPG axes are suggested, the detailed mechanisms of how TH influences pubertal onset remain unclear. We hypothesized that TH-mediated HPG regulation may be initiated by controlling the expression of GnIH, which may act on the most upstream level of the HPG axis by inhibiting the neuronal activities of GnRH and kisspeptin, and also gonadotropin secretion. Therefore, we investigated the possible role of GnIH as a novel mediator between the HPT and HPG axes. To do this, we examined the effect of abnormal thyroid status on the pubertal onset in female mice, and assessed the changes of the following HPG indicators; hypothalamic GnIH, Kiss1 and GnRH expression, pituitary LH release, and circulating E2 levels.

In hypothyroidic female mice, increased GnIH and decreased Kiss1 mRNA expression would result in decreased pituitary LH release and reduced circulating E2 levels, leading to the delayed pubertal onset, although there was no significant change in GnRH mRNA expression. Hyperthyroidic female mice had no difference in the timing of puberty onset, however more complicated hypothalamic control was observed; both inhibitory GnIH and stimulatory Kiss1 mRNA expressions were significantly decreased. Thus, these countervailing effects of two inputs to GnRH neurons may result in no change of GnRH mRNA expression. Although LH release was shown to be decreased in hyperthyroidism, E2 had a similar level to the control mice. This could be an explanation for the normal timing of pubertal onset in hyperthyroidic mice.

Our data showed that GnIH mRNA expression is changed by the thyroid status, which is increased by hypothyroidism and decreased by hyperthyroidism. However, the number of GnIH-positive cells in the DMH was not increased by hypothyroidism, and only significantly decreased by hyperthyroidism. GnIH-positive cells may not reflect actual levels of released GnIH, thus more quantitative analysis for GnIH peptide levels need to be performed. Nevertheless, it should be noted that hyperthyroidism induces reduction of GnIH mRNA expression and GnIH immunoreactivity, indicating that high concentration of T_3_ can actively suppress GnIH expression. This is further strengthened by the direct inhibitory effect of T_3_ on GnIH mRNA expression in hypothalamus explants. We also found that GnIH neurons express both TRα and TRβ, indicating that GnIH neurons could directly accept TH signals. However, TRs did not bind to the *GnIH* promoter region in either case of hypothyroidism or hyperthyroidism, thus TH may act through non-genomic action in GnIH neurons *via* plasma membrane or cytosol-located TRs^41^. Critically, chromatin modifications in the *GnIH* promoter are well consistent with the thyroid status-dependent GnIH expression changes; the activation marker H3Ac is enriched in hypothyroid-induced activated *GnIH* promoter, whereas the repression maker H3K9me3 is enriched in hyperthyroid-induced repressed *GnIH* promoter. Together, these results indicate that thyroid status alters GnIH expression by epigenetic modification of the *GnIH* promoter region.

As mentioned above, we observed no obvious changes in GnRH mRNA expression by abnormal thyroid status. Our data is contrary to the previous reports, in which altered GnRH pulsatile secretion, induced by hyperprolactinemia in hypothyroidism, leads to the delay in LH response^11^. Conversely, local increase of T_3_ in the basal hypothalamus was shown to induce GnRH release in seasonal reproductive animals^42^, however exogenous T_3_ in hypothalamic explants resulted in decreased GnRH mRNA change in our experiment. The possible reasons for these discrepancies may be caused by the differences in measurement of GnRH changes, mRNA expression *vs*. release, since mRNA expression may not be consistent with GnRH pulsatile secretion.

Kiss1 mRNA expression was decreased in both hypothyroidism and hyperthyroidism. Notably, Kiss1 mRNA levels were measured in the whole hypothalamus, including different kisspeptin neuronal populations in the arcuate nucleus (Arc) and anteroventral periventricular nucleus (AVPV). Kisspeptin neurons in the Arc are considered to generate GnRH/LH pulse and mediate the negative-feedback action of sex steroids on GnRH/LH pulse^43^, whereas AVPV kisspeptin neurons are involved in the control of GnRH/LH surge evoked by the positive-feedback of E2^44^. Thus, there may be some differences in TH effect on kisspeptin neurons between the Arc and AVPV. It should be further investigated.

Pituitary LH release was shown to be decreased in both hypothyroidism and hyperthyroidism. Hypothyroidism-induced low LH levels, even without reduction of GnRH expression, could be explained by combined effects of increased GnIH and decreased Kiss1 expression, since both neuropeptides can directly act on the pituitary^23, 45^. In hyperthyroidism, reduction of the inhibitory factor GnIH may have little impact on downstream regulation, whereas reduction of Kiss1 may dominantly contribute to decrease LH levels. The change of E2 levels is an indicator of pubertal onset, thus decreased E2 in hypothyroidic mice resulted in delayed pubertal onset. On the contrary, hyperthyroidism had no effect on E2 levels, consequently there was no differences on the timing of pubertal onset, although complicated upstream regulations were occurred at hypothalamus-pituitary levels of hyperthyroidic mice.

In this study, we showed that thyroid dysfunction alters hypothalamic GnIH expression through changing the chromatin status in *GnIH* promoter region in female mice, suggesting the novel function of GnIH as a mediator between the HPT and HPG axes. Increased GnIH expression induced by hypothyroidism may in part contribute to delayed puberty onset. This possibility was further investigated by using GnIH-KO mice. Knockout of GnIH, which is interpreted by the lacking of mediating factor to convey thyroid status, exhibited blunt effect on pubertal onset by hypothyroidism in contrast to WT, even though T_3_ levels were significantly decreased in PTU-administered GnIH-KO mice. These results indicate that GnIH is the critical factor to mediate the effect of abnormal thyroid status on pubertal onset. On the other hand, normal timing of pubertal onset in hyperthyroidism despite of significant reduction in GnIH expression could be explained by the suppression of an inhibitory factor having minimal effects on phenotypic changes. Similarly, there were no changes in the pubertal onset and LH levels when deficiency in the inhibitory factors are observed in GPR147-deficient female mice^46^ and amino acid γ-aminobutyric acid receptor-knockdown mice^47, 48^. However, it should be noted that GnIH-KO female mice seem to be delayed in pubertal onset compared to WT. The absence of inhibitory GnIH may cause continuous stimulation of GnRH which conversely leads to suppressed gonadotropin response, as indicated by the use of GnRH analog in treatment of precocious puberty^49^. Therefore, it should be further investigated the precise mechanism how knockout of GnIH influences on pubertal onset *via* the change of HPG regulators, and the use of highly specific antagonist for GPR147 would provide more detailed information about this novel physiological function of GnIH.

In conclusion, our findings suggest that GnIH is an important factor to keep the balance of TH-mediated HPG regulation for the proper pubertal development, indicating a novel function of GnIH to mediate the cross-talk between the HPT and HPG axes that contribute to proper timing of pubertal onset. This is the first evidence showing the involvement of GnIH in pubertal disorders induced by abnormal thyroid status.

## Methods

### Experimental subject

C57BL/6J strain mice (WT, Japan SLC, Shizuoka, Japan) and conditional GnIH-KO mice in the nervous system^40^ were used in this study. WT and GnIH-KO female mice were weaned from the dams on the 20th day after birth, and then housed on 12/12-h light/dark cycle with lights on at 08:00 a.m. in a temperature-controlled environment (23 °C) with *ad libitum* food and water. To induce hypothyroidism or hyperthyroidism, female mice aged 20 days were administered with 0.1% PTU (Wako, Osaka, Japan) or 0.001% T_4_ (Sigma-Aldrich Co., St Louis, MO, USA) in drinking water for 2 weeks, respectively, monitoring the changes of body weight and vagina opening. After 2 weeks of administration, mice were anesthetized with 3% isoflurane and decapitated between 9:00 a.m. and 13:00 p.m. to collect blood and brain samples. All experiments were conducted at Waseda University, and animal care and procedures were approved by the Waseda University Safety Management Rules for Biological Experiment. Experimental protocols that we used in this study do not require approval by an institutional and/or licensing committee of Waseda University.

### Measurement of circulating hormonal changes

Blood samples collected by cardiac puncture were placed at room temperature for 1 h and centrifuged at 5000 rpm for 10 min at 4 °C, then serum samples were stored at −80 °C until use. TSH, T_3_ and LH levels were quantified by Milliplex MAP kit (EMD Millipore, Darmstadt, Germany) using the rat thyroid hormone magnetic bead panel for TSH and T_3_, and mouse pituitary magnetic bead panel for LH. E2 levels were quantified by Chemiluminescence Enzyme Immunoassay kit (Immunosepc Co., Canoga Park, CA, USA).

### Immunohistochemistry

Mice were perfused through the heart with 0.1 M PBS flowed by 4% paraformaldehyde (PFA). After post-fixation and saturation with 30% sucrose, the brain was sectioned in coronal plane at 30 μm. Free-floating sections were rinsed in PBS with 0.3% Triton X-100 (PBST). The sections were blocked with 5% normal goat serum in PBST for 2 h at room temperature, then transferred into primary rabbit anti-GnIH antibody recognizing mouse GnIH^17^ at a dilution of 1:2000 for 24 h at 4 °C. After rinsing, the sections were incubated with Alexa Fluor 488 goat anti-rabbit IgG (Invitrogen, Carlsbad, CA, USA) in PBST at a dilution of 1:1000 for 2 h at room temperature. After washing, the sections were mounted with mounting medium and visualized by using a Nikon Eclipse E600 microscope equipped with Y-FL Epi-fluorescence (Nikon, Tokyo, Japan). The total number of GnIH-positive cells in the DMH per a mouse was calculated.

### Hypothalamic Culture and Quantitative Real-time PCR Analysis

Hypothalamic culture were conducted as described previously^31^. Brains from female mice aged 20 days were isolated and the whole hypothalamic regions were collected into Medium 199 (Gibco/Invitrogen) and preincubated for 1 h at 37 °C in an atmosphere of 80% O_2_ and 5% CO_2_, and nitrogen. Hypothalamic explants were then transferred to 24-well plate, with each well containing 1 ml of the medium supplemented with vehicle, 10^−8^ or 10^−7^ M of T_3_. After 3 h treatment, total RNA was extracted by using RNeasy Mini kit (Qiagen, Hilden, Germany) with DNase digestion (RNase-Free DNase Set; Qiagen). Extracted total RNA was subjected to reverse transcription (RT) using Prime Script RTase (Takara, Shiga, Japan). Quantitative real-time PCR (qPCR) was conducted by using the Thunderbird SYBR qPCR Mix (Toyobo, Osaka, Japan) and StepOnePlus Real-time PCR System (Applied Biosystems, Foster City, CA, USA) as reported previously^31, 37^. The house keeping gene GAPDH was used as an internal control to normalize each gene expression. Primer sequences are listed in Supplementary Table 1.

### Combination of immunohistochemistry and *in situ* hybridization

The distribution of TR mRNA was determined by *in situ* hybridization using a digoxigenin (DIG)-labeled antisense RNA probe, and the localization of GnIH was investigated on the same section by immunohistochemistry. Partial mouse TRα or TRβ cDNA was obtained by RT-PCR using the specific primers (Supplementary Table 1). The RT-PCR product was subcloned into pGEM-T vector (Promega, Madison, WI, USA). TR probes were labeled with DIG RNA labeling kit (Roche Diagnostics, Rotkreuz, Switzerland) according to the manufacturer’s instructions. The brain sections from female mice aged 20 days, with 30 μm thickness, were deproteinized with 10 mg/ml of proteinase K (Invitrogen), then fixed in 4% PFA. After fixation, the sections were incubated at room temperature with hybridization buffer, which contained 50% formamide (Wako), 5× saline sodium citrate (SSC) (1 × SSC = 150 mM NaCl and 15 mM sodium citrate, pH 7.0), 1× Denhart’s solution (Fluka Chemie GmbH, Buchs, Switzerland), 250 μg/ml of yeast RNA (Roche Diagnostics) and 500 μg/ml of DNA (Roche Diagnostics). After hybridization for 16 h at 72 °C in a hybridization buffer with RNA probes, the sections were rinsed in 0.2 × SSC for 2 h, and then blocked for 1 h in a solution of 0.1 M Tris (pH 8.0) and 0.15 M NaCl with 10% sheep serum. The sections were incubated with alkaline phosphatase-conjugated anti-DIG antibody (Roche Diagnostics) and rabbit anti-GnIH antibody at a dilution of 1:1000 for overnight at 4 °C. After washing, the sections were incubated for 2 h at room temperature in Alexa Fluor 488 goat anti-rabbit IgG (Invitrogen) at a dilution of 1:1000. After washing, alkaline phosphatase activity was detected by NBT/BCIP stock solution (Roche Diagnostics). All sections were visualized by using a Nikon Eclipse E600 microscope equipped with Y-EL Epi-fluorescence (Nikon).

### Chromatin immunoprecipitation assay

Hypothalamic samples from female mice induced into hypothyroidism or hyperthyroidism were subjected to chromatin immunoprecipitation (ChIP) assay by using SimpleChIP kit (Cell Signaling Technology; CST, Danvers, MA, USA), as instructed by the manufacturer’s protocol. After cross-linking with 37% formaldehyde (Sigma-Aldrich), hypothalamic tissue was disaggregated by Dounce homogenizer, and then sonicated by Bioruptor (Diagenode Inc., Denville, NJ, USA). Digested chromatin samples were immunoprecipitated with following antibodies; anti-TRα/β (Santa Cruz Biotechnology, Inc. USA, sc772), anti-acetyl Histone H3 (EMD Millipore #06-599), normal IgG (CST #2729), anti-Histon H3 (CST #4620), anti-Histone H3 tri-methyl K9 (Abcam, Cambridge, UK, ab8898). Cross-linking was reversed, and the DNA was purified. The recovered DNA was subjected to qPCR by using specific primers (Supplementary Table 1) designed to detect enrichment in the promoter region of mouse *GnIH*.

### Statistical analysis

All results are expressed as the mean ± SEM, except Figs 1a, 2c and 3a as the box-and-whisker plots with the smallest value and up to the largest (*Min to Max*). Statistical significance was assessed by using the GraphPad Prism 6 software (GraphPad Software, Inc., La Jolla, CA). As indicated in figure legend, data were analyzed by Student’s *t*-test (Figs 1a,b,c,e,f, 2b and 3b,c,e), one-way ANOVA followed by uncorrected Fisher’s LSD test (Fig. 4b and d), Tukey’s multiple comparisons test (Fig. 4a) or two-way ANOVA followed by a Bonferroni’s *post hoc* test for multigroup comparisons (Figs 2c and 6c,d). The overall ANOVA summary, *F* values and degrees of freedom for the ANOVAs, and reported possible interactions for the two-way ANOVAs are provided in the figure legends.

## Electronic supplementary material

Supplementary materials
