# Supplementary material for: Involvement of gonadotropin-inhibitory hormone in pubertal disorders induced by thyroid status
Source: Sci Rep. 2017 Apr 21;7:1042. doi: 10.1038/s41598-017-01183-8 (PMC5430760; doi:10.1038/s41598-017-01183-8)
Supplement: Supplementary file 1 — Supplementary materials [file 41598_2017_1183_MOESM1_ESM.pdf]

Supplementary Materials

Involvement of gonadotropin-inhibitory hormone in pubertal disorders induced by thyroid status

Mika Kiyohara[1,2,†], You Lee Son[1,†,*], Kazuyoshi Tsutsui[1,*]

[1]Laboratory of Integrative Brain Sciences, Department of Biology, Waseda University, and Center for Medical Life Science of Waseda University, Shinjuku-ku, Tokyo 162-8480, Japan.
[2]Department of Pediatrics, The Jikei University School of Medicine, Minato-ku, Tokyo 105-8461, Japan.
[*]Correspondence should be addressed to Y.L.S. (iac15016@kurenai.waseda.jp, youleeson@gmail.com) or K.T. (k-tsutsui@waseda.jp)
[†]These authors contributed equally to this work.

Supplementary Figure 1

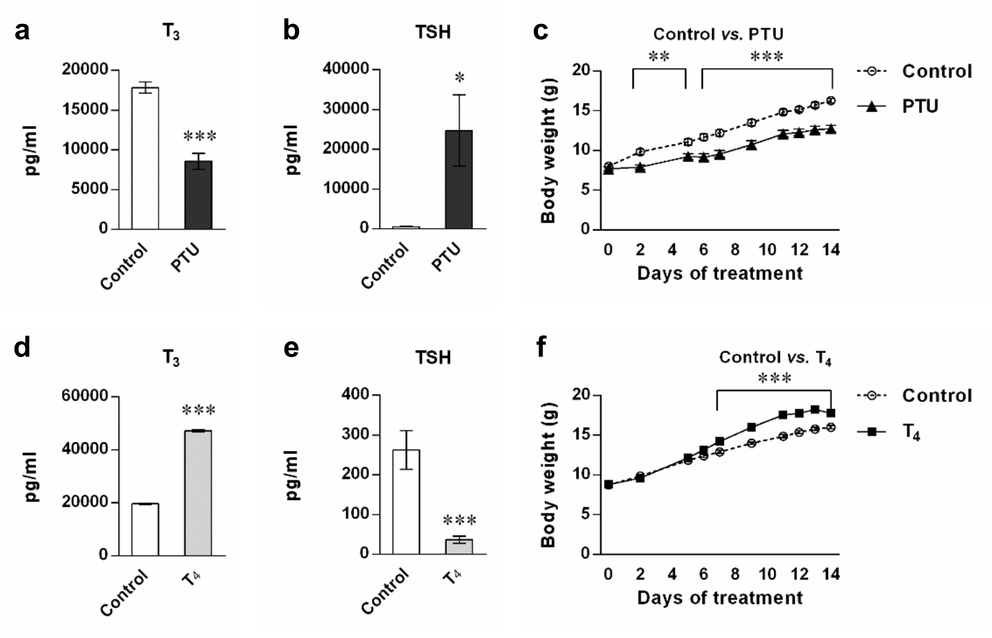

Supplementary Figure 1. Successful induction of hypothyroidism and hyperthyroidism in mice. Hypothyroidism (PTU group, Upper panel) or hyperthyroidism (T_4 group, Lower panel) was confirmed in hormonal levels of T_3 (A or D), TSH (B or E) and body weight (C or F). 0.1% PTU or 0.001% T_4 was administered to immature female mice aged 20 days in drinking water for 2 weeks, respectively. Data represent mean ± SEM (A and B, n = 7 per group; C and F, n = 10 per group, D and E, n = 6 in control and n = 7 in T_4 group). $^*p < 0.05$; $^{**}p < 0.01$; $^{***}p < 0.001$ by unpaired *t* test.

Supplementary Figure 2

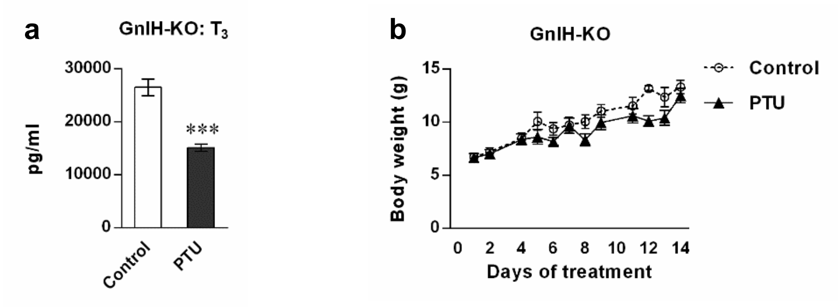

Supplementary Figure 2. Induction of hypothyroidism into GnIH-knockout female mice. Effect of PTU administration in GnIH-KO female mice. 0.1% PTU was administered to GnIH-KO female mice aged 20 days in drinking water, then the changes in T_3 levels (A) and body weight (B) were measured. Data represent mean ± SEM (B, n = 6 in WT and n = 7 in GnIH-KO group; A, n = 5 per group; B, n = 10 in control and n = 16 in PTU group). ***p < 0.001 by unpaired *t* test.

Supplementary Table 1

Target	Sequences	GenBank	Used in
**GnIH-F**	5'-TGGAAGGACCATAGATGAGAAA-3'	NM_021892.1	qPCR
**GnIH-R**	5'-GCTGTTGTTCTCCCAAACCT-3'		
**Kiss1-F**	5'-AGCTGCTGCTTCTCCTCTGT-3'	NM_178260.3	qPCR
**Kiss1-R**	5'-GCATACCGCGATTCCTTTT-3'		
**GnRH-F**	5'-AGCACTGGTCCTATGGGTTG-3'	NM_008145.2	qPCR
**GnRH-R**	5'-CCTGGCTTCCTCTTCAATCA-3'		
**GAPDH-F**	5'-ACAACTTTGGCATTGTGGAA-3'	NM_008084.2	qPCR
**GAPDH-R**	5'-GATGCAGGGATGATGTTCTG-3'		
**TRα-F**	5'-CACTCTTCCTGGAGGTCTTTGA-3'	NM_178060.2	ISH
**TRα-R**	5'-TCTATATTCTCCCCTTGCTTGG-3'		
**TRβ-F**	5'-TCCTCAGTGATGCGCTTATG-3'	NM_001113417.1	ISH
**TRβ-R**	5'-TGTGCATAAGATGGGGTCAG-3'		
- **2207F**	5'-CTGAGTGATTCCCCAAGACC-3'	NM_021892.1	ChIP-qPCR
- **2207R**	5'-TCTTGCTAGCTTGTATTATTCAGGT-3'		
- **1680F**	5'-CTGGTGGAGCAGAAGGGTAG-3'	NM_021892.1	ChIP-qPCR
- **1680R**	5'-CGCTCTAAGCAGGTCTCTGG-3'		
- **1257F**	5'-TTCTTGGCTACCACAGACCA-3'	NM_021892.1	ChIP-qPCR
- **1257R**	5'-CCCTGTTCCGAAGGTGTCTA-3'		
- **1014/999F**	5'-GTGTGGAGGTGGATGGATCT-3'	NM_021892.1	ChIP-qPCR
- **1014/999R**	5'-TTTTGGAGAGGCTGACTGGT-3'		
- **688F**	5'-GAGCAGGAAGGTAGGACGTG-3'	NM_021892.1	ChIP-qPCR
- **688R**	5'-GCAGCCTCCTTGTTATTTGC-3'		

Supplementary Table 1. Primers used in this study. Genomic sequence of GnIH promoter region was obtained from chromosome 6:50650672-50654439 bp of mouse genome.
